# Endoscopic Mini-or Less-Open Sublay Operation (E/MILOS) in ventral hernia repair: a minimally invasive alternative technique

**DOI:** 10.1590/0100-6991e-20233405-en

**Published:** 2023-03-13

**Authors:** JOÃO PAULO VENANCIO DE-CARVALHO, LUCA GIOVANNI ANTONIO PIVETTA, PEDRO HENRIQUE DE FREITAS AMARAL, EDUARDO RULLO MARANHÃO DIAS, JESSICA ZILBERMAN MACRET, HAMILTON BRASIL RIBEIRO, MAURICE YOUSSEF FRANCIS, PEDRO DE SOUZA LUCARELLI ANTUNES, WOLFGANG REINPOLD, SERGIO ROLL

**Affiliations:** 1 - Faculdade de Ciências Médicas da Santa Casa de São Paulo, Departamento de Pós-graduação em Cirurgia - São Paulo - SP - Brasil; 2 - Irmandade da Santa Casa de Misericórdia de São Paulo, Grupo de Parede Abdominal - São Paulo - SP - Brasil; 3 - Hospital Alemão Oswaldo Cruz, Centro de Hérnia - Serviço de Cirurgia do Aparelho Digestivo - São Paulo - SP - Brasil; 4 - Irmandade da Santa Casa de Misericórdia de São Paulo, Médico Assistente do Serviço de Emergência - São Paulo - SP - Brasil; 5 - Faculdade de Ciências Médicas da Santa Casa de São Paulo, Professor da Disciplina de Cirurgia - São Paulo - SP - Brasil; 6 - Irmandade da Santa Casa de Misericórdia de São Paulo, Médico Residente em Cirurgia Geral, Departamento de Cirurgia - São Paulo - SP - Brasil; 7 - Hamburg Hernia Center, Chairman and CEO - Hamburgo - Alemanha; 8 - Helios Mariahilf Hospital Hamburg, Teaching Hospital of Hamburg Medical School, Chairman of the Department of Abdominal Wall Surgery - Hamburgo - Alemanha

**Keywords:** Hernia, Ventral, Video-Assisted Surgery, Laparoscopy, Hand-Assisted Laparoscopy, Hérnia Ventral, Cirurgia Vídeoassistida, Laparoscopia, Laparoscopia Assistida com a Mão

## Abstract

The ideal ventral hernia surgical repair is still in discussion^1^. The defect closure with a mesh-based repair is the base of surgical repair, in open or minimally invasive techniques^2^. The open methods lead to a higher surgical site infections incidence, meanwhile, the laparoscopic IPOM (intraperitoneal onlay mesh) increases the risk of intestinal lesions, adhesions, and bowel obstruction, in addition to requiring double mesh and fixation products which increase its costs and could worsen the post-operative pain^3-5^. The eTEP (extended/enhanced view totally intraperitoneal) technique has also arisen as a good option for this hernia repair. To avoid the disadvantages found in classic open and laparoscopic techniques, the MILOS (Endoscopically Assisted Mini or Less Open Sublay Repair) concept, created by W. Reinpold et al. in 2009, 3 years after eTEP conceptualization, allows the usage of bigger meshes through a small skin incision and laparoscopic retro-rectus space dissection, as the 2016 modification, avoiding an intraperitoneal mesh placement^6,7^. This new technique has been called E-MILOS (Endoscopic Mini or Less Open Sublay Repair)^8^. The aim of this paper is to report the E-MILOS techniques primary experience Brazil, in Santa Casa de Misericórdia de São Paulo.

## RESUMO

O tratamento cirúrgico ideal para correção das hérnias ventrais ainda é motivo de grande discussão^1^. O fechamento do defeito associado a utilização de telas para reforço da parede abdominal são passos fundamentais da terapia cirúrgica, podendo ser realizados tanto pela via aberta quanto pelas técnicas minimamente invasivas^2^. A via aberta apresenta maiores taxas de infecção de sítio cirúrgico, enquanto o reparo laparoscópico IPOM (intraperitoneal onlay mesh) acarreta um risco aumentado de lesões intestinais, aderências e obstruções intestinais, além de requerer uso de telas de dupla face e dispositivos de fixação que encarecem o procedimento e não raro aumentam a dor no pós-operatório^3-5^. A técnica eTEP (extended/enhanced view totally extraperitoneal), tem ganhado importância, mostrando-se uma boa opção para a correção das hérnias ventrais também^2^. A fim de se evitar as desvantagens das técnicas abertas e laparoscópicas “clássicas” o conceito MILOS (Endoscopically Assisted Mini or Less Open Sublay Repair), desenvolvido por W. Reinpold et al. em 2009, 3 anos antes do advento do eTEP, possibilita ao cirurgião o uso de telas de grandes dimensões no plano retromuscular através de uma pequena incisão na pele e dissecção laparoscópica deste espaço, conforme modificação realizada em 2016, evitando a colocação de uma tela no espaço intraperitoneal^6-7^. Esta nova técnica passou a se chamar EMILOS (Endoscopic Mini or Less Open Sublay Repair)^8^ Este artigo tem como objetivo relatar nossa experiência inicial no emprego da técnica E-MILOS no Brasil, na Santa Casa de Misericórdia de São Paulo.

## DESCRIPTION OF THE TECHNIQUE AND RESULTS

According to Reinpold, the applied technique can be referred to as mini open if the skin incision is smaller than 6cm, and less open if the incision varies between 6 and 12cm, and this value must be smaller than ¼ of the largest diameter of the mesh used in abdominal wall repair.

**Figure 1:**
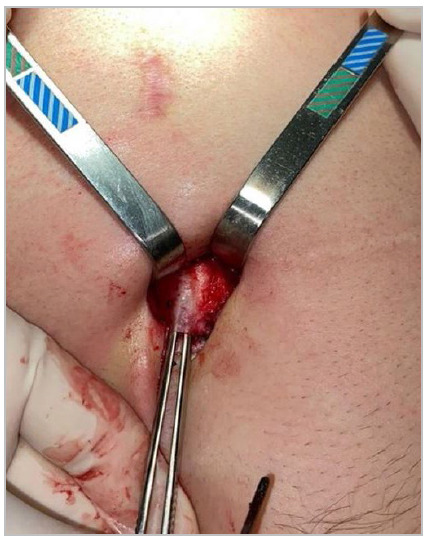
Opening of the posterior sheath of the rectus abdominis muscle.

**Figure 2:**
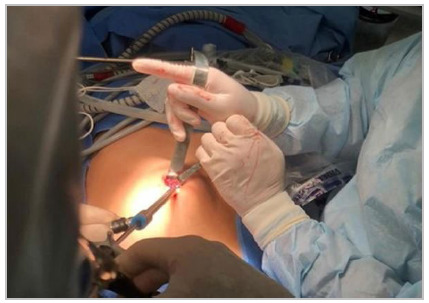
Dissection of the preperitoneal and retromuscular space with malleable retractors and the aid of the Endotorch Light Tube^TM^.

We start the procedure with an incision of about 4cm, generally periumbilical or over the hernia defect. We dissect the anterior fascia of the rectus abdominis muscle (Figure 1) and the hernia sac when present, followed by an incision at the edge of the rectus and dissection of the retromuscular space in all directions, that is, laterally, cranially, and caudally around the defect, with open surgery retractors and forceps. For the distal dissection, we use the Endotorch^TM^, which is an illuminated cannula through which we insert laparoscopy tweezers and, under direct vision, reach the most distal xiphoid and suprapubic regions (Figure 2)^6^ ^-^ ^8^.

We close the posterior fascia and introduce the Alexis^®^, which is a circular device for incision retraction that allows non-traumatic access to cavities, providing excellent exposure with a small incision. Through its lid, the first trocar is inserted, allowing the establishment of the pneumoperitoneum and the passage of the optics in the retromuscular space.

**Figure 3:**
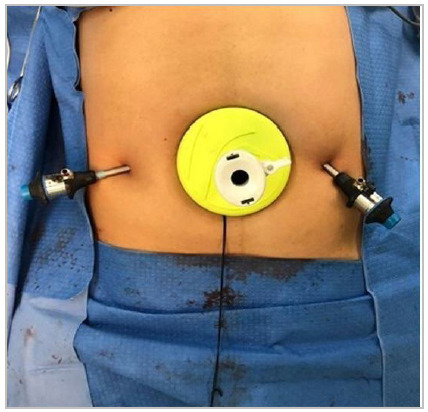
Placement of Alexis^®^ at the incision site and passage of lateral working trocars.

We proceed with the introduction of two more 5-mm lateral trocars for the laparoscopic clamps and dissection of the retromuscular space (Figure 3). This dissection must exceed at least 6 cm from the edges of the hernia defect. For this, if necessary, one can perform a posterior component separation, with the release of the transversus abdominis muscle.

**Figure 4:**
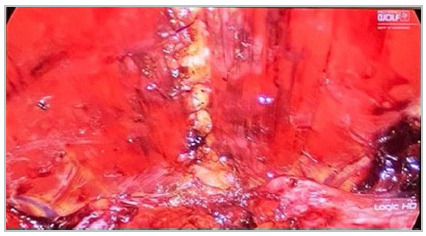
Synthesis of the defect with barbed wire associated with correction of rectus abdominis muscle diastasis

**Figure 5:**
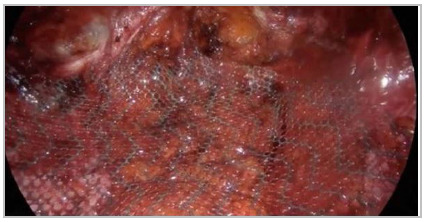
Placement of the mesh in the retromuscular space

Next, we close the hernia defect on the anterior wall with absorbable suture (Figure 4) (barbed, if available) and placement of a mid or high weight, large-pore polypropylene mesh (Figure 5). We fixate it with glue or suture and, depending on the size of the defect, we don’t fixate it at all.

Finally, we remove the trocars and the Alexis with subsequent closure of the anterior fascia of the rectus abdominis, fixation of the umbilical scar (if necessary), and skin closure.

Our initial experience with this technique involved eight patients, with a mean age of 46.6 years (range 20 - 65), six females and two males. The mean BMI was 26.4kg/m^2^ (range 18.9 - 33.8) and the main comorbidity in this group was smoking, present in 37.5% of cases.

We corrected a total of nine hernias, as one of the patients had two distinct defects in the abdominal wall. Of these, seven were incisional and one was primary (umbilical). Furthermore, only two were outside the midline. Correction of rectus abdominis muscles diastasis was performed in 37.5% of the cases. The average size of the hernias was 4.3cm (range 1.5 - 8.0) and the average size of the mesh used was 20 x 18cm (range 15 x 15 - 25 x 20). The average surgical time was 3.5 hours.

We converted one case to open surgery due to technical difficulties, and in only three cases the retromuscular space was drained, with an average drain time of 6.3 days.

The mean length of stay was 1.75 days, with a mean postoperative follow-up time of 13 months (range 1 - 21).

None of the patients operated on evolved with postoperative complications and, to date, no patient has had hernia recurrence.

Steps description

1. Periumbilical incision (infra or supra)
2. Complete dissection of the hernia sac
3. Complete dissection of the hernia ring
4. Opening of the posterior sheath of the rectus
5. Posterior fascia dissection using malleable retractors
6. Dissection of the retromuscular and preperitoneal space in the linea alba with the Endotorch Light Tube^TM^
7. Incision of the posterior sheath in a longitudinal direction in the four quadrants to the medial border of the rectus abdominis muscle
8. Placement of Alexis^®^ with lid
9. Insertion of a 10 or 12mm trocar into the Alexis^®^
10. CO_2_ insufflation (10mmHg) into the retromuscular space
11. Insertion of two 5mm trocars
12. Endoscopic dissection of the retromuscular space with preservation of the linea alba, having the xiphoid process as the cranial limit and the lateral borders of the rectum and the semilunar line as the lateral ones
13. Closure of the hernia defect (technical options: Rives-Stoppa or Posterior Components Separation)
14. Placement of polypropylene mesh
15. Alexis^®^ withdrawal
16. Closure of the anterior fascia of the rectus abdominis

## DISCUSSION

Our first results of the E/MILOS technique in the treatment of ventral hernias, both primary and incisional, are promising. The combination of the benefits of the open technique with placement of a large mesh in the retromuscular space associated with a small incision (Figure 7), which generates lower rates of postoperative complications and less trauma to the abdominal wall, provides the E/MILOS technique with the main benefits of both open and laparoscopic repairs, avoiding their main limitations ^8^.

E/MILOS should preferably be indicated in patients who need reinforcement of the entire abdominal wall in the midline topography, that is, patients who have rectus abdominis diastasis associated with a ventral hernia^9^. Despite the literature indicating the technique predominantly in cases where the hernia is in the midline, our experience shows that the application of the technique is feasible in cases outside the midline as well.

It is important to emphasize that the surgical time of more than three hours is related to the technique’s learning curve, which, according to the author of the technique, is between five and 10 procedures, depending on the surgeon’s expertise^7^.

Other advantages of the technique are listed in Table 1.

**Table 1:** Main Advantages of the E-MILOS Technique.

1.	Hybrid minimally invasive technique
2.	Placement of large meshes in the retromuscular space
3.	No need to fixate the mesh in all cases
4.	Less postoperative and chronic pain
5.	Ease of closing the defect
6.	Ease of hernia sac dissection and peritoneum closure
7.	Ease of umbilicus reconstruction
8.	Fewer skin incisions compared with eTEP
9.	Allows minimally invasive treatment of rectus diastasis, off-midline hernias, concomitant ventral hernias, and large ventral hernias with the aid of posterior component separation

## CONCLUSION

When compared with open surgery with mesh placement in the retromuscular space or with laparoscopic techniques (IPOM and eTEP), E/MILOS is as effective as the traditional techniques for correcting ventral hernias, but with a significantly lower number of postoperative complications, reoperations, and unplanned readmissions. In addition, we confirmed that the technique is easily reproducible and feasible in a public hospital.
